# Availability of family planning services and quality of counseling by faith-based organizations: a three country comparative analysis

**DOI:** 10.1186/s12978-017-0317-2

**Published:** 2017-05-08

**Authors:** Janine Barden-O’Fallon

**Affiliations:** 1 Carolina Population Center, University of North Carolina at Chapel Hill, Chapel Hill, NC USA; 2 Department of Maternal & Child Health, Gillings School of Global Public Health, University of North Carolina at Chapel Hill, 400 Meadowmont Village Circle, 3rd Floor, Chapel Hill, NC 27517 USA

**Keywords:** Family planning, Faith-based organizations, Malawi, Kenya, Haiti

## Abstract

**Background:**

Faith-based organizations (FBOs) have a long history of providing health services in developing countries and are important contributors to healthcare systems. Support for the wellbeing of women, children, and families is evidenced through active participation in the field of family planning (FP). However, there is little quantitative evidence on the availability or quality of FP services by FBOs.

**Methods:**

The descriptive analysis uses facility-level data collected through recent Service Provision Assessments in Malawi (2013–14), Kenya (2010), and Haiti (2012) to examine 11 indicators of FP service and method availability and nine indicators of comprehensive and quality counseling. The indicators include measures of FP service provision, method mix, method stock, the provision of accurate information, and the discussion of reproductive intentions, client’s questions/concerns, prevention of sexually transmitted infections, and return visits, among others. Pearson’s Chi-square test is used to assess the selected indicators by managing authority (FBO, public, and other private sector) to determine statistical equivalence.

**Results:**

Results show that FBOs are less likely to offer FP services than other managing authorities (*p* < 0.05). For example, 69% of FBOs in Kenya offer FP services compared to 97% of public facilities and 83% of other private facilities. Offering long-acting or permanent methods in faith-based facilities is especially low (43% in Malawi, 29% in Kenya and 39% in Haiti). There were few statistically significant differences between the managing authorities in comprehensive and quality counseling indicators. Interestingly, Haitian FBOs often perform as well or better than public sector health facilities on counseling indicators, such as discussion of a return visit (79% of FBO providers vs. 68% of public sector providers) and discussion of client concerns/questions (52% vs. 49%, respectively).

**Conclusions:**

Results from this analysis indicate that there is room for improvement in the availability of FP services by FBOs in these countries. Quality of counseling should be improved by all managing authorities in the three countries, as indicated by low overall coverage for practices such as ensuring confidentiality (22% in Malawi, 47% in Kenya and 12% in Haiti), discussion of sexually transmitted infections (18%, 25%, 17%, respectively), and providing services to youth (53%, 27%, 32%, respectively).

## Plain English summary

Faith-based organizations (FBOs) have a long history of providing health services in developing countries and are important contributors to healthcare systems. Support for the wellbeing of women, children, and families is evidenced through active participation in the field of family planning (FP). However, there is little evidence on the availability or quality of FP services by FBOs. The objective of this research is to investigate the provision of FP services by FBOs in Malawi, Kenya, and Haiti. Data collected from health facilities is used to examine 11 measures of FP service and method availability and nine measures of FP counseling quality according to the managing authority- faith-based, public, or other private sector provider. Findings show that faith-based facilities are less likely to offer FP services than facilities of other managing authorities. Offering or providing long-acting or permanent methods in faith-based facilities is especially low. There were few significant differences in comprehensive and quality counseling between the managing authorities. Interestingly, Haitian faith-based facilities often perform as well or better than public sector health facilities on assessed measures. This analysis contributes information on a topic which has very little evidence. Results from this analysis indicate that there is room for improvement in the availability of FP services by FBOs in these countries. Quality of counseling should be improved by all managing authorities in the three countries.

## Background

Faith-based organizations (FBOs) have a long history of providing health services in developing countries and are important contributors to healthcare systems. With a mission to serve their communities, especially the poor and other marginalized groups, FBOs are particularly well-positioned to contribute to health care delivery. In many countries, FBOs supplement government health services, contribute resources such as buildings, equipment, and staff from a wide range of donors, and are well-integrated in the communities they serve [1]. However, the actual degree to which FBOs contribute to healthcare has not been adequately quantified. In a first attempt to measure religious contributions to health systems, the African Religious Health Assets Program estimated approximately 40% of national health services in Lesotho and 30% in Zambia were provided by religious entities [2]. An average of “about 40% in many sub-Saharan African nations” was then reported in a seminal World Health Organization report [1] and has since been widely cited as the estimated contribution of FBOs in healthcare services [3, 4]. A more recent attempt to clarify and quantify the contribution of FBOs to healthcare delivery in developing countries found wide variation, ranging from 4 to 44% for the assessed health care indicators [5]. The study’s authors acknowledge that a large gap remains in knowledge and data on this issue [5]. A review of research on faith-based health care in Africa published in 2015 echoes this finding [6]. Furthermore, there is a scarcity of evidence on the quality of healthcare services by FBOs as compared to those found in the public or other private sector [6]. One exception is a systematic review of literature from six African countries, which found that maternal and newborn services provided by FBOs were similar to those offered by the public sector, while client reported satisfaction and quality were better [7].

FBOs have an inherent interest in improving the wellbeing of the women, children, and families for whom they serve and are active in the field of family planning (FP) and reproductive health. Faith entities support various elements of FP as essential for reducing maternal and child morbidity and mortality, eradicating poverty, and minimizing or eliminating induced abortion; education and other social welfare programs are also understood to benefit from support of FP [8]. FP is accepted by many Christian and Muslim organizations and leaders [9]: over 1,000 religious leaders have endorsed a commitment to safe, affordable, accessible and comprehensive FP services [10]. With a high level of credibility and trust within their communities, FBOs influence reproductive health choices and behavior, and have the standing to influence decisions on FP at all levels, from the individual to the policy level [11].

Despite such broad support for family health and FP in particular, there are noteworthy areas in which FBOs and secular health services can differ. For example, there may be religious-based objections to the provision of certain FP services or contraceptive methods. This would be the case for organizations supported by the Catholic Church, for example, which officially supports only natural FP methods such as the Standard Days Method, TwoDay Method, and Lactational Amenorrhea Method. FBOs may also object to offering FP services to certain types of clients, such as unmarried adolescents, whose sexual behavior would be forbidden by religious teachings. Indeed, religious barriers against modern contraception are cited as reasons for individual non-use of contraception. For example, among Kenyan women not intending to use contraception in the future, 9% report a religious prohibition as their reason for future non-use [12]. A study from Pakistan reported 22% of respondents were not using contraception due to religious concerns [13].

The availability and accessibility of FP services and an array of contraceptive methods are key elements of global FP policies. The global FP initiative started in 2012, FP2020, has a goal of providing 120 million women and girls with modern contraceptives by 2020 [14]. To help achieve this goal, the FP2020 Rights and Empowerment Working Group has established common principles for each of ten dimensions of FP [14]. Availability and service quality are two of the ten dimensions [14]. Regarding availability, the principles state that health care facilities, providers and contraceptive methods need to be available “to ensure that individuals can exercise full choice from a full range of methods” and that furthermore, contraceptive methods are to be accessible without informational or other barriers [14]. Regarding service quality issues, the principles state that “client-provider interactions respect informed choice, privacy and confidentiality, client preferences, and needs” [14]. A core partner of FP2020 is the United States Agency for International Development (USAID), which is also the world’s largest FP bilateral donor [15]. USAID-supported FP programs are guided by principles of volunteerism and informed choice, meaning that programs are required to provide information on a wide variety of FP choices, including the benefits and health risks of particular methods, and that a wide variety of methods are offered [16]. The Livingston Amendment ensures that FBOs providing natural FP methods are supported as long as they offer information about or access to other methods through referral [16].

Currently, there is insufficient information on the degree to which faith-based facilities support method availability, volunteerism and free choice on a range of methods, and how FBOs compare to other FP providers on these issues [17]. The objective of this research is to investigate the provision of FP services by FBOs in three countries, Malawi, Kenya, and Haiti. These three countries were chosen for analysis based on the availability of recent health facility data and the active participation of FBOs in the healthcare sector. Specifically, the research will examine the availability of FP services and methods and the quality of counseling in FBOs as compared to public and other private sectors in the three selected countries.

## Methods

This is a descriptive analysis conducted for three countries with recent Service Provision Assessments (SPA), carried out by the Demographic and Health Survey program, and includes Malawi, Kenya, and Haiti [18–20]. The SPA use four data collection tools, including a facility inventory questionnaire, health provider interviews, provider-client observations, and exit interviews with clients to collect comprehensive data on a number of key health service indicators, including availability, readiness, quality, and client satisfaction. SPA data are publically available and cover a variety of key health services in addition to FP, such as child health, maternal and newborn health, HIV/AIDS, and malaria. Written consent for interviews and oral permission to observe consults were obtained at the time of survey administration. Survey protocols were reviewed by the ICF International IRB and in-country IRBs and comply with the U.S. Department of Health and Human Services regulations for the protection of human subjects. Malawi, Kenya and Haiti were selected based on the availability of recent SPA data and sufficient representation of FBOs in the data.

The Malawi SPA was conducted in 2013–14. The data are from a census of facilities; of a total of 1,060 facilities, 977 (92%) completed all data collection tools and are included in the dataset. The Kenya SPA was conducted in 2010 and provides a nationally representative sample of the country’s health facilities, including all national and provincial hospitals, which covers approximately 11% of the total population of the country’s health facilities. Hospitals, maternity and stand-alone voluntary, counseling and testing facilities were oversampled; sampling was not conducted on the basis of managing authority. The Haiti SPA was conducted in 2012. Like Malawi, it is a census of facilities with 905 of 907 total facilities included in the dataset. Further details on SPA data and data collection can be found at http://dhsprogram.com/What-We-Do/Survey-Types/SPA.cfm [21].

For this analysis, all facilities were classified by their managing authority as either 1) faith-based; 2) public sector; or 3) other private sector (includes private for-profit and private not-for-profit). Eleven indicators for FP service and method availability were selected, and include:

1. Percent of facilities offering any FP services
2. Percent of facilities offering at least 2 modern methods
3. Percent of facilities offering at least 4 modern methods
4. Percent of facilities offering any long acting or permanent method: intrauterine contraceptive device (IUCD), implant, female sterilization, or male sterilization
5. Percent of facilities providing any FP services
6. Percent of facilities providing at least 2 modern methods
7. Percent of facilities providing at least 4 modern methods
8. Percent of facilities providing any long acting or permanent method: IUCD, implant, female sterilization, or male sterilization
9. Number of days per week facility offers FP services (categorized 1–2, 3–4, 5+)
10. Percent of facilities with method provided available on day of assessment for combined oral contraception pills (“pills”), progesterone-only injectable, IUCD, and all provided methods. At least one method must be observed and valid for use to qualify.
11. Percent of facilities with routine user fees or charges/routine fees for FP services

The indicators for availability reflect those presented in the SPA reports, covering the areas of method mix and specific methods offered, frequency of services, and stock availability [18–20]. An indicator on fees for services was added to reflect an additional potential barrier to services. In the SPA surveys, “a facility is said to *offer* a FP method if the facility reports that it provides the method, prescribes the method for clients to obtain elsewhere, or counsels clients on the method without actually making that method available to the client in the facility. A facility is said to *provide* a family planning method if the facility reports that it stocks the method and makes it available to clients when they visit the facility. In other words, these clients can obtain the method without leaving the facility” ([18], p.66). Though this terminology can be somewhat pedantic, it provides useful information on whether facilities are offering a full range of methods even when they are not delivering them, due to facility size, lack of appropriately trained providers, or other reasons. Abortion is not included as a FP service in the SPA.

Nine quality of counseling indicators were chosen using the Quick Investigation of Quality (QIQ), a validated tool designed to assess the quality of family planning services [22]. Of the 25 recommended indicators in the QIQ, those that were specifically related to provider’s interpersonal skills, provision of information, and client’s method choice, and that were included in the SPA data, were selected for analysis. An additional indicator for services to youth was added.

1. Percent of providers who looked at and wrote on client’s health card
2. Percent of providers who used any visual aids
3. Percent of providers who discussed a return visit
4. Percent of providers assuring client of confidentiality
5. Percent of providers asking clients about reproductive intentions. Indicator includes providers asking clients about the desire for a(nother) child and/or the desired timing for birth of the next child.
6. Percent of providers discussing questions/concerns with client on current method. Indicator includes if provider asked client if he/she had questions or concerns regarding current method, or if client expressed concerns about method or asked questions about method, including the possible side effects of method.
7. Percent of providers discussing client’s perceived risk of sexually transmitted infections (STI/HIV), use of condoms to prevent STI/HIV, or dual method use
8. Percent of providers giving accurate information on the method accepted. Indicator is a composite of three items: provider explains how to use method, discusses possible side effects, and what to do if side effects.
9. FP provider provides services to youth (FP provider offers “youth friendly” or services to adolescents). Services to adolescents or youth friendly services are defined as services “designed to be youth friendly with the specific aim of encouraging youth or adolescent utilization.”

The indicators focus on availability of services and comprehensive quality of counseling and do not measure other aspects of service provision, such as technical competence (i.e. training or supervision) or facility readiness (i.e. supplies or infection control). Data for the availability indicators come from the facility audit, while counseling indicators use data from observations, client exit interviews, and provider interviews. Pearson’s Chi-square test was used to assess the frequency of the selected indicators across each type of managing authority (FBO, public sector, and other private sector) to determine statistical equivalence. Results of the multiple category comparisons that are statistically significant at *p* < 0.05 are indicated in the tables. Analyses are weighted for sample design (Kenya) and non-response (Malawi; Haiti) to compensate for any over- or under- representation of facility type in the data, and use weights provided in each dataset. Sample sizes are not weighted in order to present actual numbers used in the final samples.

### Settings

#### Malawi

The Christian Health Association of Malawi is a health service network of independent church-affiliated facilities, located mostly in rural areas [18]. The Association is co-owned by the Malawi Council of Churches and the Episcopal Conference of Malawi; about half of its facilities are Catholic and half Protestant. The Christian Health Association of Malawi facilities provide the vast majority of faith-based service provision in Malawi and are a key element of the healthcare system.

Malawi is a mostly Christian country, though Muslims make up a considerable portion of the population: 21% of Malawians report being Catholic, 65% as other Christian, and 13% as Muslim [23]. Generally, Muslims tend to have higher fertility rates than non-Muslims, and this is the case in Malawi: Muslims have a total fertility rate of 7.0 as compared to 5.3 for Catholics, and 5.6 for other Christians [23]. Contraceptive use also tends to be lower for Muslims (32%) than for non-Muslims (48%) [23]. Overall, 42% of married women age 15–49 are current modern contraceptive users, with the most widely used methods being injectables (26%) and female sterilization (10%) [24]. Among current modern method users, 9% of women in Malawi report receiving their method at a facility managed by the Christian Health Association of Malawi, compared to 74% of women receiving a method from a public facility, and 17% from other private facilities [24].

#### Kenya

Three main faith networks provide health services in Kenya. The Kenya Conference of Catholic Bishops is the largest faith-based health network and the umbrella organization for all Catholic hospitals and clinics. The network only provides natural FP methods. The second-largest faith-based health network is the Christian Health Association of Kenya, which is the umbrella organization for all Protestant hospitals and clinics. The Supreme Council of Kenya Muslims is also an active network in health service provision.

Religious affiliations in Kenya are similar to those in Malawi: 22% are Catholic, 68% are other Christian, and 7% are Muslim [23]. Fertility is higher for Muslims than non-Muslims (a total fertility rate of 5.1 compared to 4.5) and contraceptive use is lower (20% compared to 48%) [23]. Modern contraceptive use in Kenya is the highest among the three countries in this study, at 53% among currently married women age 15–49 [12]. The most common methods are injectables (26%) and implants (10%) [12]. Only about 2% of women report getting their method at a faith-based church, mission hospital or clinic, as compared to 60% from the public sector, 31% from other private, and 6% from “other” sources [12].

#### Haiti

There is no uniting health care network operating in Haiti, though many religious organizations are involved in health service delivery, including for example, IMA World Health, Catholic Medical Mission Board, and International Child Care. Demographic and Health Survey data show that 53% of women and 45% of men belong to a Protestant denomination, while 39% of women and 42% of men are Catholic; 7% of women and 12% of men report having no religion [25].

The modern contraceptive prevalence rate among married women age 15–49 in Haiti is 31%, with injectables (19%) and the male condom (5%) as the most common methods [25]. Unlike Malawi and Kenya, the public sector is not the most common source of contraception: 30% of women report the private sector as their source of method, followed by the public (23%), “mixed” (14%), and non-institutional, non-medical and “other” (34%) [25]. The source of method by FBO is not reported in the country’s Demographic and Health Survey.

## Results

### Distribution of facilities by managing authority

Of the 167 faith-based facilities included in the Malawi SPA, 160 belong to the Christian Health Association of Malawi. All health facilities managed by FBOs in Malawi represent 17% of the sample, which compares to 48% by the public sector and 35% by the other private sector (see Table 1). The majority of faith-based facilities are health centers (66%), followed by hospitals (26%). This pattern is more similar to public facilities than other private facilities, which are mostly clinics (87%).

**Table 1:** Percentage of faith-based, public and other private sector facilities in Malawi, Kenya, Haiti; MSPA 2013–14, KSPA 2010, HSPA 2012

	Malawi				Kenya				Haiti			
	FBO (*n* = 167)	Public (*n* = 478)	Other Private (*n* = 332)	TOTAL (*n* = 977)	FBO (*n* = 93)	Public (*n* = 347)	Other Private (*n* = 255)	TOTAL (*n* = 695)	FBO (*n* = 183)	Public (*n* = 342)	Other Private (*n* = 380)	TOTAL (*n* = 905)
Type of Facility												
Hospital	26	10	6	12	9	9	5	7	7	15	15	13
Health center	66	72	5	48	16	17	3	11	15	14	14	14
Dispensary	1	9	2	5	63	72	14	49	NA	NA	NA	NA
Clinic	6	4	87	34	10	2	71	29	42	51	28	40
Other^a^	1	5	0	2	2	1	7	3	37	21	42	33

*NA* Not applicable. Column totals of over 100% are due to rounding

^a^Malawi = Health post; Kenya = Maternity, Voluntary counseling & testing centers; Haiti = Health center without beds

The Kenya SPA data show that faith organizations manage 13% of health facilities in Kenya, as compared to the public sector, at 50%, and other private sector, at 37%. In Kenya, the majority of faith-based facilities are dispensaries (63%), which is also the most common facility type in the public sector (72%). The other private sector facilities are most likely to be clinics (71%).

In Haiti, the SPA data indicate that FBOs manage 20% of health facilities, as compared to 38% by the public sector and 42% by all other private sector. The most common faith-based facility type are clinics (42%), followed by health centers without beds (37%). Both of these facility types are the most common in the public and other private sector as well.

### Availability of services

Tables 2, 3 and 4 present results for the eleven indicators of availability of services and methods by managing authority. Assessment of the results by country shows that in Malawi (Table 2), there are many statistically significant differences in the availability of FP services and methods by managing authority. 57% of faith-based facilities offer FP services, compared to 95% of public and 77% of other private facilities (*p* < 0.05). Most facilities offering FP services offer at least four modern methods. Overall, 69% of all facilities in Malawi offer a long acting method, such as an IUCD, implant, or female or male sterilization, with 43% of faith-based facilities offering such methods (*p* < 0.05). However, the provision of long acting methods (i.e. providing the methods on-site) is lower on average, at 58%, and 32% for faith-based facilities (*p* < 0.05). Faith-based facilities have a routine user fee in 98% of facilities; this is also a common practice in other private facilities (80%), but not in the public sector (8%) (*p* < 0.05). Almost two-thirds of faith-based facilities are open five or more days/week, which is less than other private facilities (85%) but compares favorably to the public sector (49%) (*p* < 0.05). Finally, having valid stock of popular methods such as pills and injectables is relatively common for all facilities, but less so for the IUCD (68% overall, 37% in faith-based facilities) (*p* < 0.05). Having all methods provided in stock on the day of the interview occurred for 42% of all facilities, with no statistical difference by managing authority.

**Table 2:** Percentage of faith-based, public, and other private sector facilities with family planning services available in Malawi; MSPA 2013-14

	FBO (*n* = 167)	Public (*n* = 478)	Other Private (*n* = 332)	TOTAL (*n* = 977)
Availability of Service Indicators				
Offers any FP services	57	95	77	83**
Offers at least 2 modern methods	56	95	76	82**
Offers at least 4 modern methods	54	93	69	78**
Offers any of: IUCD, implant, female or male sterilization	43	88	55	69**
Provides any FP services	57	95	77	82**
Provides at least 2 modern methods	56	94	74	81**
Provides at least 4 modern methods	48	89	59	71**
Provides any of: IUCD, implant, female or male sterilization	32	78	43	58**
Facility has routine user fees or charges (Malawi, Haiti); routine fees for family planning services (Kenya)	98	8	80	48**
# of days per week facility offers FP services				(*n* = 810)
1-2 days	35	42	14	32**
3-4 days	2	9	1	5**
5+ days	63	49	85	62**
Method stock on day of assessment				(*n* = varies by method)
Provides pills with pills available	80	90	79	86**
Provides injectable with injectable available	89	90	88	89
Provides IUCD with IUCD available	37	55	80	68**
Every method provided available	40	41	45	42

** Significant at *p* < 0.05

**Table 3:** Percentage of faith-based, public, and other private sector facilities with family planning services available in Kenya; KSPA 2010

	FBO (*n* = 93)	Public (*n* = 347)	Other Private (*n* = 255)	TOTAL (*n* = 695)
Availability of Service Indicators				
Offers any FP services	69	97	83	88**
Offers at least 2 modern methods	44	96	83	85**
Offers at least 4 modern methods	44	95	75	81**
Offers any of: IUCD, implant, female or male sterilization	29	58	51	51**
Provides any FP services	41	96	81	83**
Provides at least 2 modern methods	41	96	78	82**
Provides at least 4 modern methods	39	94	64	76**
Provides any of: IUCD, implant, female or male sterilization	18	38	37	35
Facility has routine user fees or charges (Malawi, Haiti); routine fees for family planning services (Kenya)	58	68	92	76**
# of days per week facility offers FP services				(*n* = 573)
1-2 days	21	11	11	12
3-4 days	0	0	0	0
5+ days	79	89	88	88
Method stock on day of assessment				(*n* = varies by method)
Provides pills with pills available	100	96	88	94
Provides injectable with injectable available	100	97	91	95
Provides IUCD with IUCD available	98	98	81	92**
Every method provided available	80	84	64	77**

** Significant at *p* < 0.05

**Table 4:** Percentage of faith-based, public, and other private sector facilities with family planning services available in Haiti; HSPA 2012

	FBO (*n* = 183)	Public (*n* = 342)	Other Private (*n* = 380)	TOTAL (*n* = 905)
Availability of Service Indicators				
Offers any FP services	89	93	72	84**
Offers at least 2 modern methods	86	93	68	81**
Offers at least 4 modern methods	79	88	59	74**
Offers any of: IUCD, implant, female or male sterilization	39	50	29	39**
Provides any FP services	82	91	62	77**
Provides at least 2 modern methods	78	90	56	73**
Provides at least 4 modern methods	63	70	41	56**
Provides any of: IUCD, implant, female or male sterilization	21	27	16	21**
Facility has routine user fees or charges (Malawi, Haiti); routine fees for family planning services (Kenya)	98	94	92	94**
# of days per week facility offers FP services				(*n* = 753)
1-2 days	2	3	6	4**
3-4 days	4	1	7	4**
5+ days	94	96	87	92**
Method stock on day of assessment				(*n* = varies by method)
Provides pills with pills available	88	90	85	88
Provides injectable with injectable available	90	89	89	89
Provides IUCD with IUCD available	25	56	64	55
Every method provided available	62	61	55	59

** Significant at *p* < 0.05

Results for Kenya (Table 3) show that 69% of faith-based facilities offer FP services, as compared to 97% of public and 83% of other private sector facilities (*p* < 0.05). The availability of methods shows a large difference by managing authority in Kenya: 44% of faith-based, 95% of public, and 75% of other private facilities offer at least four modern methods; this drops for offering any long acting method: only 29% of faith-based facilities offer such methods, as compared to 58% of public and 51% of other private facilities (*p* < 0.05). Provision of long acting methods is low for all types of managing authority (35%). Routine user fees are less common in faith-based facilities (58%) than in the public (68%) or other private (92%) sector (*p* < 0.05). Most facilities in Kenya that offer FP services are open five or more days a week (88%) and have stock on hand for pills (94%) and injectables (95%). Faith-based facilities are almost as likely to have all methods provided on hand as public sector facilities (80% vs. 84%).

Of the three countries, the highest percentage of faith-based facilities offering FP services is found in Haiti (89%) (Table 4). Many faith-based facilities offer four or more modern methods (79%), though only 39% offer any long acting method (compared to 50% of public and 29% of other private) (*p* < 0.05). The provision of long acting methods by health facilities in Haiti is low (21%), though faith-based facilities are slightly more likely than other private facilities (16%) to provide such methods (*p* < 0.05). In fact, for many of the availability indicators, faith-based facilities exhibit a pattern of higher coverage than other private facilities and lower coverage than public sector facilities. Routine user fees are common across all managing authorities (92–98%) and most facilities offer FP services five or more days/week (92%) (*p* < 0.05). Like Malawi and Kenya, stock is more likely to be on-hand for popular short-term methods, but IUCD was only on-hand for 55% of facilities (though very few facilities offer this method to begin with (*n* = 31)). Almost 60% of facilities had all methods provided available on the day of the interview.

### Comprehensive & quality counseling

Results for the nine indicators on comprehensive and quality counseling are shown in Table 5. Overall, there are fewer statistically significant differences by managing authority in these indicators than in the group of availability indicators. In Malawi, the percentage of providers in faith-based facilities writing on health cards and discussing a return visit is higher than the percentage of providers in public or other private facilities performing these skills (*p* < 0.05). However, providers in faith-based facilities do not perform as well on the other indicators, and are less likely to assure clients of confidentiality (15% vs. 22% average) and discuss dual method use and STI/HIV risk or prevention (8% vs. 18% average) (*p* < 0.05).

**Table 5:** Percentage of faith-based, public, and other private sector facilities with comprehensive and quality family planning counseling in Malawi, Kenya, and Haiti; MSPA 2013–14, KSPA 2010, HSPA 2012^a^

	Malawi				Kenya				Haiti			
	FBO (*n* = 156)	Public (*n* = 1101)	Other Private (*n* = 282)	TOTAL (*n* = 1539)	FBO (*n* = 86)	Public (*n* = 817)	Other Private (*n* = 122)	TOTAL (*n* = 1025)	FBO (*n* = 316)	Public (*n* = 667)	Other Private (*n* = 324)	TOTAL (*n* = 1307)
Comprehensive & Quality Counseling Indicators												
Provider Practices												
Looked at and wrote on client health card	96	94	89	94**	91	95	94	95	46	44	42	44
Used any visual aids	21	26	19	25	25	24	29	25	10	9	6	9
Discussed a return visit	95	87	88	88**	97	97	95	97	79	68	79	74**
Assured client of confidentiality	15	22	29	22**	58	47	40	47	15	11	13	12
Asked clients about reproductive intentions	21	27	29	26	21	24	28	24	7	5	3	5
Discussed client concerns/questions about current method	69	77	72	76	70	79	90	80	52	49	56	51
Discussed dual method use, STI/HIV risk or prevention	8	19	17	18**	26	26	19	25	15	14	23	17**
				(*n* = 1468)								(*n* = 1270)
Gave accurate information on use, side effects and what to do if problems occur with use	39	49	50	48	56	50	52	51	36	46	45	44**
				(*n* = 2262)				(*n* = 1625)				(*n* = 1947)
Offers services to adolescents/“youth friendly” services	50	52	28	53	28	31	18	27**	31	25	40	32**

** Within country comparisons significant at *p* < 0.05

^a^Data from observations, client exit interviews, and provider interviews

The selected counseling indicators do not significantly vary by managing authority in Kenya, with the exception of the provision of youth friendly-services. Though the indicator shows low coverage (27%), providers in faith-based facilities and public facilities are more likely to provide such services (28% and 31%, respectively) than are other private providers (18%) (*p* < 0.05).

Providers in faith-based facilities in Haiti are slightly more likely to write on client health cards (46%) than are providers in facilities of other managing authorities (average 44%), though the difference is non-significant, and are more likely to discuss a return visit (79%) compared to public sector providers (68%) (*p* < 0.05). Other indicators with significant variation by managing authority include the discussion of dual method use and STI/HIV risk or prevention, the provision of accurate information on use of the method, and the provision of youth friendly services. On the selected quality of counseling indicators, Haitian faith-based facilities often perform better than public facilities and the same as or lower than other private facilities. Four comprehensive and quality counseling indicators had an average coverage less than 20%.

## Discussion

This analysis compared the performance of faith-based facilities against public and other private facilities for a number of indicators on availability of FP services and quality of counseling. The analysis was conducted for three countries with recent SPA data. Results of this analysis show that FBOs manage between 13 and 20% of the health facilities in these countries; depending on the volume of clients in the facilities, these figures may or may not result in a provision of healthcare that is the “average of 40%” often attributed to FBOs in sub-Saharan Africa and developing countries [1]. Faith-based facilities in these countries occupy a service provision space that is similar to the public sector: while the type of facility most commonly managed by FBOs varies by country (health centers in Malawi, dispensaries in Kenya, and clinics in Haiti), these are also the most common types of facilities for the public sector in each of these countries.

The availability of FP services by faith-based facilities varied by country. For example, the percent of faith-based facilities offering FP services in Malawi was 57% compared to 89% in Haiti. However, faith-based facilities in the three countries generally lagged behind the other types of managing authority in indicators associated with FP service availability. This is especially true for the offer/provision of long acting methods. With the exception of Haiti, which actually performed better than public sector facilities on these indicators, faith-based facilities in Malawi and Kenya were much less likely to offer or provide long acting methods. Offering or providing at least four modern methods was also low for faith-based facilities in Kenya (44% and 39% respectively). Upholding principles of volunteerism and the ability to freely choose methods requires that information about a range of methods is available. While not all facilities are equipped to provide long acting methods, all should be able to provide information and counseling on method options and referrals, especially if there are no religious restrictions to offering such methods. Improvements in service availability can be made in Malawi, Kenya and Haiti if more FBOs offer FP services, offer and provide a range of methods that includes long acting methods, and ensure method stock.

Comprehensive and quality counseling also varied by country, and by indicator, though there were fewer statistically significant differences between the managing authorities. This was especially the case in Kenya. Some indicators were relatively high for all managing authorities. For example, provider’s discussion of client concerns/questions was fairly common in the three countries, with no statistical differences between managing authorities. Conversely, use of visual aids during counseling and provider’s discussion of client’s reproductive intentions was not common for any managing authority in any of the countries, and hardly occurred at all in Haitian facilities. There was also generally low coverage for assuring client confidentiality (22% in Malawi, 47% in Kenya and 12% in Haiti), and discussing dual method use and perceived risk of STI/HIV (18%, 25%, 17%, respectively); these practices were particularly uncommon for FBOs in Malawi (15% and 8%, respectively). Approximately one-third of facilities in Kenya (27%) and Haiti (32%) and slightly more than half of facilities in Malawi (53%) offer services to adolescents or “youth friendly” services, which suggests that the youth in these countries likely experience barriers to access FP services.

The percentage of providers in FBOs performing the assessed counseling indicators was often the lowest of the three managing authorities in Malawi; this was not the case in Kenya or Haiti. In fact, while the quality of counseling indicators show low coverage overall in Haiti, the performance of providers in faith-based facilities is generally better than the performance of providers in public sector facilities and sometimes better than that of the other private sector facilities. This may be due in part to intense work by FBOs to restore and improve the provision of health care after the earthquake in 2010 [26]. Because quality of care is key to the mission of serving communities and improving wellbeing, it is an area for potential strengthening among FBOs in Malawi.

The main strength of this analysis is that it uses recent, nationally representative data collected from health facilities. The data can be analyzed by managing authority, thus allowing for an analysis of services by the faith-based health sector. This analysis is therefore able to contribute information on a topic, the provision of health services by FBOs, which suffers from a lack of quantitative evidence. Moreover, the analysis is noteworthy in its focus on FBOs and FP services. This type of information is essential to inform faith networks, donor partners, policymakers, and other FP stakeholders, on potential service gaps and areas for future collaboration to strengthen the availability and provision of FP services to meet FP2020 goals. The analysis highlights two important elements of FP service delivery, the availability of services and methods and counseling, which may be of particular relevance for faith-based health care networks. The analysis also expands the evidence base by including a country from the Latin America and Caribbean region. A significant limitation of this analysis is that the specific religious affiliation of the FBOs is not collected in the SPA. As a result, information is lost by analyzing FBOs as one category, especially in countries such as Kenya, with many religions and denominations providing health care services, some with more restrictions than others. This is an important limitation as previous research has noted that heterogeneity among religious teachings can have fundamental effects on the provision of health care [17]. While the SPA is able to make comparisons across broad types of managing authorities, a modification to the SPA data collection tools to allow categorization of faith-based facilities by religion and denomination would greatly enhance research on this topic. In addition, facility-based surveys developed with the specific intent to assess variation in faith-based care are needed. Information derived from such surveys would be of great benefit to other service sectors looking to improve health market efficiencies and fill gaps where needed. Additionally, there are other aspects of FP service provision that were beyond the scope of the study. These include facility readiness and provider technical competency, as well as other indicators for service quality, such as treating the client with respect and having acceptable waiting times. It is not known how FBOs would perform on these other indicators.

## Conclusions

FBOs have the potential to be important contributors to successful FP efforts. Identified facilitators for strengthening partnerships between faith-based and secular FP programs include emphasizing mutual goals, respecting philosophical differences, and including the faith community in the development of national FP strategies [9]. A diversity of partnership approaches may be necessary [8]. However, national FP programs need to ensure that volunteerism and free choice are upheld, which means ensuring access to information on a wide range of methods and maintaining choice for a wide range of methods, regardless of the service provider. Additional research is needed to better understand the role of FBO’s in the provision of FP services in developing countries, especially by religion and denomination. Results from this analysis indicate that there is room for improvement in the availability of FP services by FBOs in these countries. Quality of counseling, especially with practices such as assuring client confidentiality, asking clients about their reproductive intentions, and discussing dual method use and STI/HIV risk or prevention should be improved by all managing authorities in the three countries, and particularly by FBOs in Malawi.

## Abbreviations

FBOs: Faith-based organizations
FP: Family planning
IUCD: Intrauterine contraceptive device
SPA: Service provision assessment
STI/HIV: Sexually transmitted infections/human immunodeficiency virus
USAID: United States Agency for International Development
